# Gallstone Ileus with COVID‐19 Infection: An Unintended Sequela of Non‐Operative Management of Acute Cholecystitis During the COVID‐19 Pandemic

**DOI:** 10.1002/ccr3.4275

**Published:** 2021-07-19

**Authors:** Adil A. Shah, Shai Stewart, Ahmed Raza, Wasay Nizam, Mikael Petrosyan, Mallory Williams, Edward E Cornwell

**Affiliations:** ^1^ Department of Surgery Howard University Hospital and College of Medicine Washington DC USA; ^2^ Department of Surgery George Washington University School of Medicine Washington DC USA

**Keywords:** acute care surgery, complicated cholecystitis, gallstone ileus, small bowel obstruction

## Abstract

Appropriate risk stratification and careful follow‐up are mandated in elderly patients with comorbidities. Herein, we report a case presenting 5 months after the nonoperative management of acute cholecystitis during the height of the COVID‐19 pandemic.

## Graphical abstract

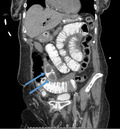

## 1 INTRODUCTION

Gallstone ileus is a rare complication occurring in approximately 0.5% of patients with cholelithiasis. It constitutes 0.1% of all mechanical bowel obstructions. The condition typically occurs in elderly patients in the 6th to 8th decade of life with a high preponderance in women.^1^ The likelihood of developing a gallstone ileus is naturally higher after an episode of untreated cholecystitis.^1^, ^2^ The resulting gallbladder wall inflammation coupled with the pressure necrosis produced by large gallstones leads to erosion of these stones through the gallbladder and enteric walls producing a cholecystoenteric fistula and subsequent passage of the stone into the small bowel lumen causing a distal obstruction. These fistulae more commonly occur between the gallbladder and the duodenum.^3^, ^4^ Herein, we report the case and long‐term sequelae of a patient with gallstone ileus who was initially treated nonoperatively with antibiotics during the peak of the COVID‐19 pandemic.

## 2 CASE HISTORY/EXAMINATION

An 87‐year‐old African‐American lady, a resident of a nursing home, presented with a 3 days history of nausea, intractable bilious emesis especially with food intake, anorexia, and inability to pass stool. This was accompanied by epigastric abdominal pain that was severe and cramping in nature. She denied a previous episode. She denied any fevers, chills, cough, or difficulty breathing. Her medical comorbidities consisted of hypertension, type 2 diabetes, osteoarthritis, hyperlipidemia, and dialysis‐dependent end‐stage renal disease. She reported a history of a hysterectomy 10 years ago.

Of note is a history of admission to the hospital in March 2020 with fever and shortness of breath attributed to influenza A infection, with concomitant right upper quadrant. The ultrasound performed at the time demonstrated‐echogenic foci noted in the gallbladder associated with shadowing, consistent with cholelithiasis associated with thickened gallbladder wall and minimal pericholecystic fluid collection. She was diagnosed with acute cholecystitis; however, given the improvement in symptoms and normalization of her white cell count, surgical intervention was deferred and the patient was treated with antibiotic therapy alone.

On this admission, vitals demonstrated tachycardia between 110 bpm and 150 bpm and systolic blood pressure ranging from 90 to 120 mm Hg. Her physical examination revealed a soft abdomen without distention. She was found to be tender in the upper abdomen with a maximal point of tenderness in the epigastrium and the periumbilical area on the abdomen. Consistent with her remote history of a hysterectomy, a well‐healed right paramedian scar was noted. Stool was noted in the rectal vault on digital rectal examination.

## 3 DIFFERENTIAL DIAGNOSIS, INVESTIGATIONS, AND TREATMENT

**FIGURE 1:**
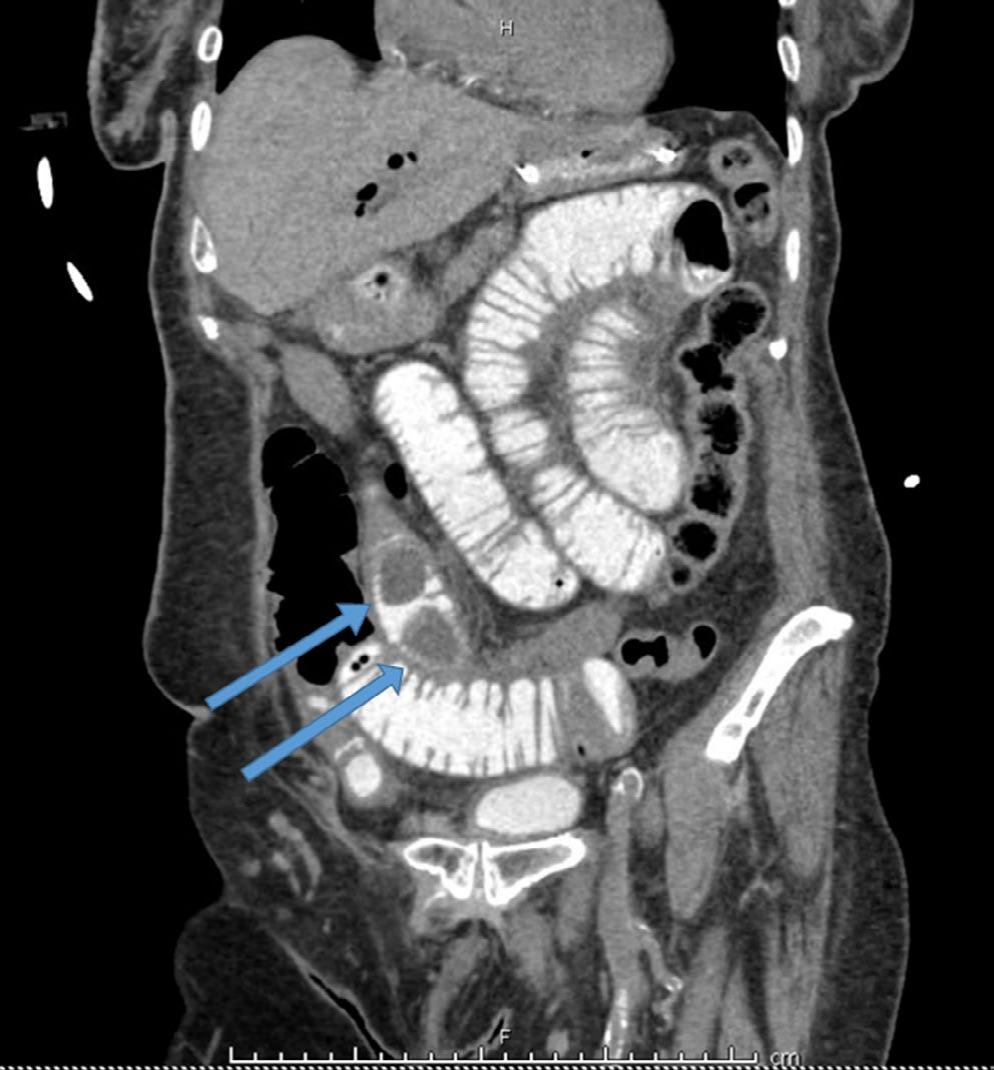
Coronal view of a computed tomography scan of the abdomen and pelvis (enhanced with oral contrast). Arrows point to filling defects resulting from gallstones in the small‐bowel lumen

Laboratory workup demonstrated a normal white cell count with a leftward shift, and hypokalemic, hypochloremic metabolic alkalosis. Her liver function tests were within normal limits. A COVID‐19 screening test performed on admission was positive for COVID‐19 infection. She was admitted to the surgical intensive care unit for management of an adhesive small bowel obstruction likely adhesive in nature due to her previous surgery. Fluid resuscitation was begun to address the metabolic alkalosis, and electrolyte replacement therapy was administered via intravenous route. Follow‐up laboratories demonstrated a corrected potassium level of 3.9 mEq/L (Milliequivalents per liter). Simultaneously, a nasogastric tube was placed. After a period of decompression, gastrografin was administered via the nasogastric tube and a CT Abdomen and pelvis were obtained after 6 hours of per‐oral contrast administration. This demonstrated two filling defects in the small bowel with no contrast passage past the second filling defect along with pneumobilia (Figure 1). These appeared to be radiolucent gallstones on close examination. Given the presence of pneumobilia, dilated small bowel loops, and gallstones in the small bowel (Rigler's triad), the diagnosis of gallstone ileus was made, and the patient was taken for operative intervention.

## 4 OUTCOME AND FOLLOW‐UP

**FIGURE 2:**
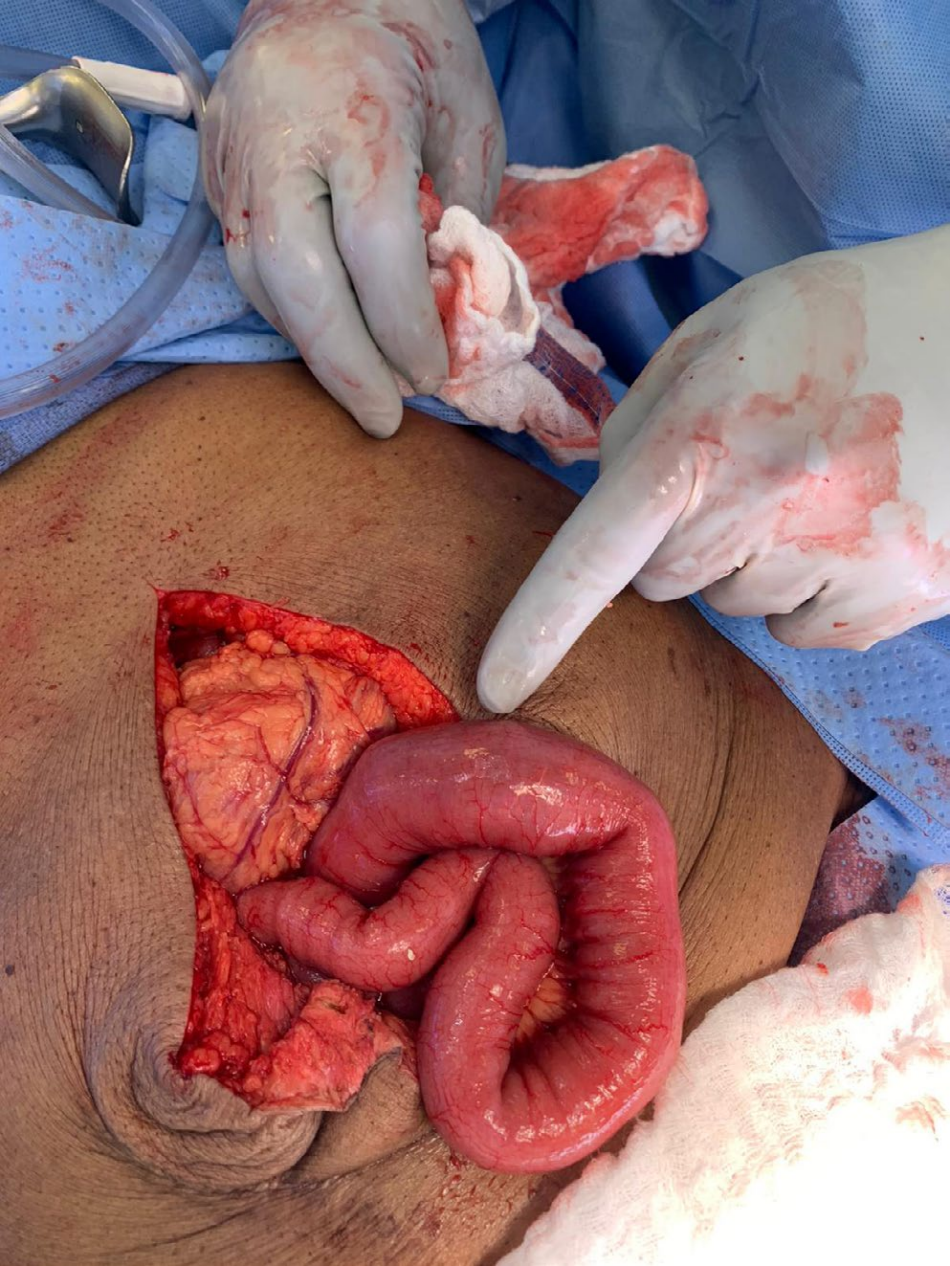
Intraoperative demonstration of obstructed bowel

**FIGURE 3:**
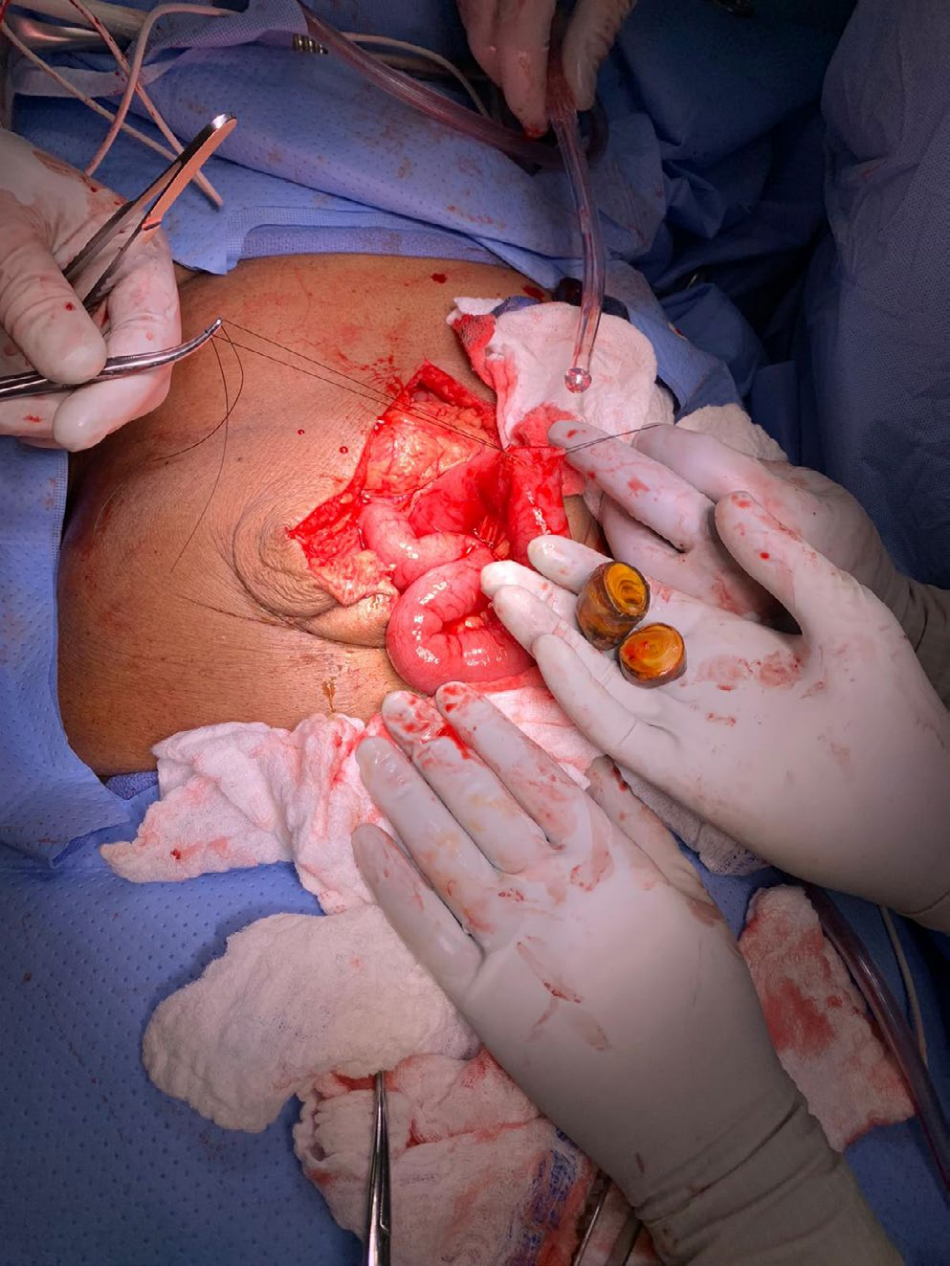
Extracted gallstones measuring approximately 3 × 3 cm and 3 × 2 cm

**FIGURE 4:**
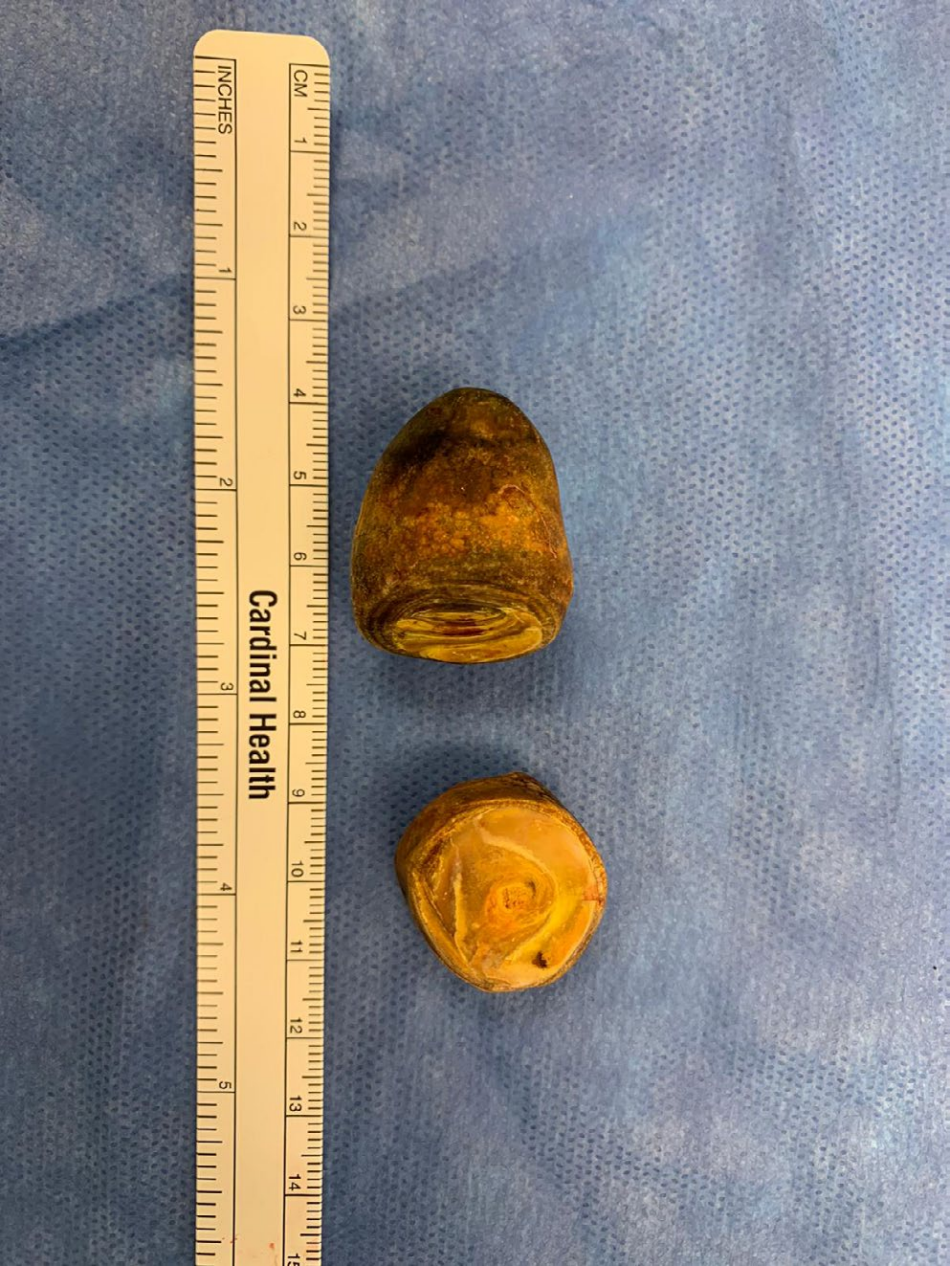
Gross view of extracted gallstones measuring approximately 3 × 3 cm and 3 × 2 cm

In the operating room, an upper midline incision was made. There were minimal adhesions encountered. The small bowel was examined from the ileocecal valve to the ligament of Treitz and the area of obstruction was easily identified at mid‐jejunum (Figure 2). 3‐0 Silk stay sutures were placed laterally. A transverse incision was made proximal to the area of obstruction, and 2 gallstones were extracted measuring approximately 3 × 3 cm and 3 × 2 cm (Figures 3 and 4). The enterotomy was then closed in two layers.

Attention was then diverted to the right upper quadrant to expose the gallbladder fundus. Significant dense adhesions were encountered. Lysis of adhesions was attempted to expose the fundus as much as possible for the extraction of two residual stones in the gallbladder lumen via a fundic incision, without taking down the cholecystoenteric fistula. This fistula was noted to connect the inferior peritoneal surface of the gallbladder to the third part of the duodenum and was surrounded by a dense inflammatory reaction. Given the presence of significant adhesions to the hepatic flexure and the duodenum, the risks of further dissection were thought to outweigh the benefits and this was aborted. This concluded the exploration. The fascia was approximated, and the patient was extubated and transferred to the surgical intensive care unit in stable condition. Her postoperative course was unremarkable, and she was successfully discharged back to her nursing home on postoperative day 7. She was followed in the outpatient setting and displayed excellent wound healing with no perioperative complications noted. A detailed discussion was held with the patient's family to discuss the role of further procedures, such as a completion cholecystectomy, to appropriately prevent any future recurrences. Considering the overall health of the patient, an eventual decision was made against a potentially morbid reoperation. The patient's caregivers and family were provided extensive counselling and education to help recognize future worrisome signs that may suggest a possible recurrence. Follow‐up evaluation at 10 months was also unremarkable.

## 5 DISCUSSION

Gallstone ileus is a mechanical intestinal obstruction due to gallstone impaction within the gastrointestinal tract. Less than 1% of cases of intestinal obstruction are derived from this etiology. The term “ileus” is a misnomer since the obstruction is a true mechanical phenomenon. Surgical relief of gastrointestinal obstruction remains the mainstay of operative treatment.

Gallstone ileus has shown a constant incidence of 30­35 cases/10 00 000 admissions over 45 years.^5^ It constitutes the etiologic factor in less than 5% of cases of intestinal obstruction ^6^ elderly patients. The most frequent fistula occurs between the gallbladder and the duodenum, due to their proximity.^7^, ^8^ Once the gallbladder is free of calculi, it may become a blind sinus tract and contract down to a small fibrous remnant. The majority of gallstones smaller than 2‐2.5 cm may pass spontaneously through a normal gastrointestinal tract and will be excreted uneventfully in the stools.^1^, ^7^, ^8^ Impacted gallstones range in size from 2 to 10 cm, with a mean of 4.3 cm.^9^

In 1941, Rigler et al described four radiographic signs in gallstone ileus: (1) partial or complete intestinal obstruction; (2) pneumobilia or contrast material in the biliary tree; (3) an aberrant gallstone; and (4) change of the position of such gallstone on serial films. The presence of two of the three first signs has been considered pathognomonic and has been found in 20%‐50% of cases.^1^ Although pathognomonic, reports of Rigler's triad range from 0% to 87%. Computed tomography (CT) is considered superior to plain abdominal films or ultrasound in the diagnosis of gallstone ileus cases, with a sensitivity of up to 93%. The frequency of Rigler's triad detection is higher with CT. In a retrospective study by Lassandro et al, Rigler's triad was observed in 77.8% of cases utilizing CT, compared to 14.8% with radiographs and 11.1% with ultrasound.

The main therapeutic goal was the relief of intestinal obstruction by the extraction of the offending gallstone. Fluid and electrolyte imbalances and metabolic derangements due to intestinal obstruction, delayed presentation, and pre‐existing comorbidities are common and require management before surgical intervention.^1^

There is no consensus on the indicated surgical procedure. The current surgical procedures are as follows: (1) simple enterolithotomy; (2) enterolithotomy, cholecystectomy, and fistula closure (one‐stage procedure); and (3) enterolithotomy with cholecystectomy performed later (two‐stage procedure). The main long‐standing controversy in the management of gallstone ileus is whether biliary surgery should be carried out at the same time as the relief of obstruction of the bowel (one‐stage procedure), performed later (two‐stage procedure) or not at all.

In 2003, Doko et al reported a 30‐patient series with morbidity of 27.3% in patients undergoing enterolithotomy alone and 61.1% for a one‐stage procedure. Mortality was 9% following enterolithotomy and 10.5% after a one‐stage procedure. American Society of Anesthesiologists (ASA) scores were similar between the two groups, but operating times were significantly longer for the one‐stage procedure. Urgent fistula repair was significantly associated with postoperative complications. The authors concluded that enterolithotomy is the procedure of choice, with a one‐stage procedure reserved for patients with acute cholecystitis, gallbladder gangrene, or residual gallstones.^10^ Similar results were also seen in a large database study performed by Mallipeddi et al^11^ where simultaneous cholecystectomy at the time of operation was noted to increase rates of minor complications, increased operative time, and longer hospital stays with no decrease in mortality, leading the authors to conclude that a simple enterolithotomy may be reasonable in challenging cases.

Many patients with gallstone ileus are elderly, with comorbidities, in poor general condition and have a delayed diagnosis, leading to dehydration, shock, sepsis, or peritonitis. Relief of gastrointestinal obstruction by simple enterolithotomy is likely the safest procedure for these patients. However, this approach theoretically does leave the risk of future recurrent episodes, as the fistulous tract remains in situ. This risk has been estimated to be approximately 5%‐8%.^12^, ^13^ Various treatment modalities have been described to address recurrent gallstone ileus. A recent algorithm by Rabie et al^14^ proposed performing a repeat enterolithotomy for unstable patients. Additionally, they proposed either an additional cholecystectomy with fistula repair for stable patients with good accessibility or for those with moderate inflammation, hindering appropriate access, an enterolithotomy with cholecystolithotomy. The United States detected its first case of the novel coronavirus disease 2019 (COVID‐19) on 20 January 2020. A surge in cases combined with system issues such as a shortage of personal protective equipment, healthcare workers, and institutions surpassing their bed capacity has greatly impacted the delivery of patient care especially during the peak of the pandemic. Additionally, recent data demonstrated that a perioperative diagnosis of COVID‐19 conferred a 24% 30‐day mortality and a 51% risk of post‐op pulmonary complications. These pulmonary complications resulted in a 38% 30‐day mortality and accounted for 83% of deaths.

This report highlights the unintended sequelae of nonoperative management of a largely surgical disease during the height of the COVID‐19 pandemic. The patient's pre‐existing conditions and COVID‐19 infection increased her risk of morbidity and mortality; however, careful perioperative optimization and a deliberately short operative time prevented undue complications.

## CONFLICT OF INTEREST

None declared.

## AUTHOR CONTRIBUTIONS

AAS made substantial contributions to conception, design, data acquisition, and interpretation of the study; involved in initial drafting of the manuscript; and critically revised the final manuscript. SS, AR, WN, and MP made substantial contributions to conception and design, of the study; involved in initial drafting of the manuscript; and critically revised the final manuscript. MW and EEC made substantial contributions to conception and design of the study, acquisition, analysis, and interpretation of data; was involved in initial drafting of the manuscript; and critically revised the final manuscript for important intellectual content.

## ETHICAL APPROVAL

Written informed consent for publication of this case report was obtained from the patient.

## CONSENT STATEMENT

Written informed consent was obtained from the patient's family prior to submission.

## Data Availability

Data sharing not applicable to this article as no datasets were generated or analysed during the current study.
